# Energy drink consumption among Israeli‐Arab adolescents: Gender differences in anxiety and well‐being

**DOI:** 10.1002/puh2.187

**Published:** 2024-07-17

**Authors:** Lili Nimri, Bshara Mansour, Amir Benhos, Abdallah Banna, Elias Nasrallah, Marwan Sackran, Ahlam Abu Ahmad, Ziv Ardi, Omer Horovitz

**Affiliations:** ^1^ Nutrition Department Tel‐Hai Academic College Kiryat Shmona Israel; ^2^ Azrieli Faculty of Medicine, Pediatrics Department, Saint Vincent de Paul Hospital Bar‐Ilan University Ramat Gan Israel; ^3^ Psychology Department Tel‐Hai Academic College Kiryat Shmona Israel; ^4^ Department of Behavioural Sciences Kinneret Academic College on the Sea of Galilee Galilee Israel

**Keywords:** adolescents, anxiety, energy drinks, mental health, well‐being

## Abstract

**Background:**

Israeli‐Arab adolescents are reported to consume higher amounts of fast food, sweets, and candies. This study examined possible associations among energy drink consumption, anxiety, and well‐being in Israeli‐Arab adolescents. We also investigated these associations concerning fast food consumption, physical activity, and hours of sleep per night.

**Methods:**

A cross‐sectional exploratory study assessed adolescents’ energy drink consumption, nutritional and lifestyle habits, well‐being, and anxiety using self‐reported questionnaires. Adolescents aged 12–18 from several cities and villages in northern Israel were considered for the study. Anthropometric measurements—weight, height, and waist circumference—were also assessed.

**Results:**

One hundred and fourteen adolescents (59 females) participated in the study. Gender and energy drink consumption were associated with psychological status. Energy drink consumers reported a lower well‐being index and a higher anxiety index than nonconsumers (73.02 ± 2.64 and 23 ± 1.56 vs. 79.37 ± 1.67 and 18.86 ± 1.41, respectively). Energy drink consumers ate significantly more fast food per week than nonconsumers (1.25 ± 0.07 vs. 1.03 ± 0.09). Additionally, hours of sleep per night were correlated with anxiety levels among energy drink consumers [*r*_s_ = 0.352, *p* = 0.018]. These results differed between genders.

**Conclusions:**

Our study shows complex associations between energy drink consumption and mental health indices. These associations can serve as a basis for further research into this topic.

## Graphical abstract

There is a link between nutrition and mental health. A study involving 114 Arab Israeli adolescents (59 females) aged 12–18 explores associations among energy drink consumption, fast food consumption, anxiety, and well‐being. Results suggest gender differences and highlight the need for further research into these complex relationships.

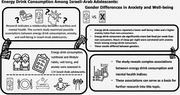

## INTRODUCTION

The nutritional psychology field suggests that healthy and balanced nutrition can affect several processes related to psychological disorders [1]. In young and adult subjects, these associations have been studied for specific disorders (e.g., depression, anxiety, attention disorders, schizophrenia, and the onset of psychotic attacks) [2, 3, 4]. However, despite evidence of such associations, results are inconsistent [5, 6]. Studies have shown that specific dietary patterns and nutrient intakes can influence mental health outcomes [7]. Eicosapentaenoic acid, docosahexaenoic acid, alpha‐tocopherol, magnesium, and folic acid demonstrated beneficial effects on stress, sleep disorders, anxiety, mild cognitive impairment, and neuropsychiatric disorders. These findings underscore their positive role in preserving normal brain function and promoting mental well‐being [8]. However, the multifaceted nature of psychological disorders introduces complexity to the interpretation of these findings. Individual variations in genetics, lifestyle factors, and the heterogeneous nature of mental health conditions contribute to the variability in research outcomes. This underscores the need for further investigation to elucidate the connections between nutrition and psychological well‐being [7]. The diverse methodologies employed in different studies, from self‐reported dietary assessments to controlled interventions, further emphasize the intricate challenge of establishing consistent associations in nutritional psychology [9, 10].

Data on associations between nutritional habits and diets and psychological symptoms in Israel are scarce. Moreover, only a few studies and surveys have compared the nutritional habits, diseases, and mental health of the various ethnic groups characterizing the Israeli population (i.e., Jews and Arabs) [11]. In a survey by the Israeli Ministry of Health 2011 on the Arab society in Israel, out of 1192 participants, 59.2% reported that family members consume energy drinks (ED) in general. It also showed that 38.9% of the children in these families consume energy drinks. Thus, 42.3% of 636 Arab adolescents aged 16–18 years consume energy drinks, 35.7% consume one can of energy drinks each time, 5.2% consume more than one can each time, and 1.4% less than one can [12]. In a survey of 14–18‐year‐old Jewish adolescents in Tel Aviv, among 802 participants, 30% reported consuming one can or less of energy drinks a day [13]. The Israeli Ministry of Health's “Mabat” youth survey (2015–2016) revealed a difference in the prevalence of obesity between Israeli Arab and Jewish youth (definition according to the World Health Organization—age‐ and sex‐adjusted body mass index (BMI) percentile ≥97%); with 18.2% for boys and 10.5% for girls among Israeli Arabs and 10.7% for boys and 8.6% for girls in the Jewish sector. It was found that Israeli Arab youth consume more fast food per week, more sweets and candies (daily), and more total sugar (g/day) than Jewish youths [14, 15]. Data from these surveys suggest that the daily consumption of different nutritional ingredients (e.g., saturated fat, caffeine, and sugar) differs between the Arab and Jewish youth sectors [12, 13, 14, 15]. Surveys have reported that the incidence of deaths from chronic diseases, such as diabetes, hypertension, and cardiovascular diseases, in the Israeli‐Arab population is 2.25‐, 1.85‐, and around 1.6‐fold higher, respectively, than in the Jewish sector [16]. These data strengthen and support research to enhance our understanding of the Arab sector's behavior and nutritional choices.

It is accepted that a “traditional/Mediterranean” diet characterized by consuming vegetables, fish, olive oil, whole grains, and legumes is related to a decrease in affective symptoms of depression and anxiety. In contrast, the “Western diet” characterized by consuming processed grains, saturated fat, red meat, sugar, and fast foods is related to increased affective symptoms [6, 17]. Energy drinks have become popular recently and fall under the “unhealthy” diet [18]. Consumption of energy drinks has been linked to various physiological and psychological issues, including hypertension, liver damage, and psychotic states [19]. Given the popularity of energy drinks among adolescents, the adverse effects encompass psychological and physiological aspects. Adolescents who regularly consume energy drinks exhibit increased odds of experiencing daily headaches, sleeping problems, irritation, tiredness, and late bedtimes [20]. Moreover, energy drinks consumption in adolescents is significantly associated with an increased prevalence of depression symptoms, heightened emotional difficulties, and lower general subjective well‐being [21]. A review paper further emphasizes the association between energy drinks consumption in adolescents and engaging in risky behaviors, such as sensation‐seeking and heightened anxiety [22]. These findings underscore the importance of raising awareness about the potential risks of energy drinks consumption and its adverse psychological outcomes among adolescents.

Given these data, an assessment of the nutritional habits—and especially energy drinks consumption—of Israeli Arab adolescents and their possible relation to the adolescents’ psychological status is warranted. Although energy drinks consumption by adolescents is distressing, this period of life is also challenging for implementing nutritional practices and lifestyle changes. Thus, focusing on this age group might promote an understanding of all spheres' short‐ and long‐term impacts, including health, behavior, and education. The current study evaluates the association among adolescents’ energy drinks consumption, lifestyle, and nutritional habits (physical activity, hours of sleep per night, and fast‐food consumption) with psychological parameters (anxiety and well‐being).

## METHODS

### Study design, population, and sampling

This cross‐sectional study on psychological indices and nutritional habits of Israeli‐Arab adolescents aged 12–18 from several northern Israel cities and villages was conducted between August 2019 and March 2020. Participants were recruited based on age, were generally healthy, and did not suffer from any chronic disease. The study employed a random sampling method with a demographically diverse sample, ensuring representativeness and minimizing potential selection.

To determine the sample size needed to examine the associations among energy drinks consumption, psychological status, nutritional and lifestyle habits, and well‐being among Israeli‐Arab adolescents, we used a power calculation that drew effect sizes based on effects obtained in studies conducted on energy drinks consumption in adolescents (*d* = 0.25) [19, 22, 23]. The sample size was calculated using G*power computer software, and based on analysis of variance (ANOVA), main effects and interactions, a medium effect size (*f* ^2^ = 0.27), *α* = 0.05, 80% power, and four covariates, a sample size of 110 subjects was needed. We expected that 10% of the acquired data would not be usable. Thus, we aimed to recruit more than 110 participants.

### Study variables and data collection

Our study focuses on psychological indices and nutritional habits, and the exclusion criteria have been defined to eliminate factors that may influence these measurements, ensuring the precision and reliability of our findings; these criteria included gastrointestinal diseases, diabetes, coronary diseases, traumatic brain injury, psychological disorders, and eating disorders. A registered dietitian/nurse recorded height, weight, and waist circumference at the hospital; a link was sent to the participants’ cell phones, where they filled out all questionnaires (completion of all questionnaires was estimated at approximately 20 min). The adolescents completed the questionnaires independently, without the presence of their parents, to minimize potential bias.

#### Anthropometric profile

Height (to ±0.1 cm) was measured using a stadiometer. Bodyweight (to ±100 g) was measured on a precision scale in the morning with light clothes and no shoes after voiding bowels and bladder. For the determination of BMI (kg/m^2^), weight (kg) was divided by the square of height (m). Weight was categorized based on the WHO weight classification based on BMI‐for‐age *z*‐scores for children and adolescents (5 through 19 years old) [24]. Additionally, waist circumference was determined with a tape measure. Waist circumference and height data were also used to calculate waist‐height ratio (WHtR), where WHtR above 0.5 is associated with a higher risk of metabolic complications in children and adolescents [25]. One dietitian/two nurses (always the same ones) performed all tests, repeated each twice, and then calculated the average.

#### The screen for child anxiety‐related disorders (SCARED)

We used the 41‐item child self‐reported screen for child anxiety‐related disorders (SCARED) questionnaire to measure anxiety symptoms. This questionnaire includes five factors in line with the previously described DSM‐IV(Diagnostic and Statistical Manual of Mental Disorders‐IV) classification of anxiety disorders [26]. The response items were “Not True or Hardly Ever True = 0,” “Somewhat True or Sometimes True = 1,” and “Very True or Often True = 2.” The range of possible scores is 0–82, and evidence suggests that scores equal to or greater than 25 effectively discriminate between anxious and non‐anxious adolescents [26]. Previous studies point to the SCARED to be reliable in a sample of clinically referred youths with reasonable child–parent agreement [27]. In the current study, the internal consistency of the SCARED questionnaire was satisfactory—*α* = 0.889.

#### Personal well‐being index: school children (PWI‐SC)

We used the personal well‐being index: school children (PWI‐SC)*—*a unidimensional and multi‐item instrument developed by Cummins and Lau [28], to measure personal well‐being in school‐aged children and adolescents. It uses an 11‐point bipolar scale and is designed for self‐administration. On this scale, “zero means you feel VERY SAD. 10 means you feel VERY HAPPY, and the middle of the scale is 5, which means you feel NOT HAPPY OR SAD.” This instrument comprises seven items corresponding to satisfaction, as previously described. Generally, higher scores reflect positive mental health. The internal consistency of the questions of the *PWI‐SC* questionnaire in the current study was satisfactory—*α* = 0.766.

#### Nutritional status questionnaire

The nutritional status questionnaire includes three sections. The first contains five sociodemographic questions. The second comprises 7 questions regarding dietary and lifestyle habits, and the third has 15 questions about energy drinks consumption habits. The questionnaire was compiled based on the “Mabat” youth survey questionnaire [14] and a literature review of energy drinks consumption [13, 19, 29]. The questions based on the “Mabat” youth survey questionnaire were originally in Arabic. The questions regarding energy drinks consumption were originally in English. It was translated from English into Arabic and then back‐translated into English by a different translator to ensure its meaning remained the same.

The first part of the survey included sociodemographic variables such as gender, age, religion, and parental level of education. In the second part of the survey, participants were asked about their nutritional and lifestyle habits, such as the frequency of eating fast food per week, physical activity per week, and hours of sleep per night. In the third part of the survey, participants were asked questions about energy drinks consumption habits, such as if they ever drank and, if so, how many cans per week they consumed. The internal consistency of the 22 questions within the two content sections used to assess nutritional and lifestyle habits and energy drinks consumption habits in the current study was satisfactory—*α* = 0.71.

#### Lifestyle and nutritional habits

To test for differences in weekly fast‐food consumption, we divided the participants’ responses into two dichotomized conditions (fast‐food consumption index): eating fast food three or fewer times a week and eating fast food four or more times a week (a similar division was proposed in a study in 2021 [30]). It was pointed out that adolescents playing sports for an hour three times per week will likely lead to a “sedentary” lifestyle [31]. Hence, to test for differences in weekly physical activity, we queried the participants for the weekly number of times they participated in physical activity. Responses were obtained on a Likert scale and were reversed to be consistent with other scales collected, ranging from 1 = 0–1 times a week, 2 = 2–3 times a week, 3 = 4–6 times a week, and 4 = every day. To test for possible associations between anxiety and well‐being levels and hours of sleep per night, we queried the participants for their hours of sleep per night on a Likert scale ranging from 1 = less than 6 h per night, 2 = 7 h per night, 3 = 8 h per night, and 4 = more than 9 h per night.

### Data analyses

The study's primary outcomes encompass three key domains: energy drinks consumption, psychological status, and nutritional and lifestyle habits. Energy drinks consumption is dichotomized into consumers and nonconsumers. Psychological status is gauged through continuous variables, precisely measuring anxiety and well‐being. Nutritional and lifestyle habits are explored through continuous and categorical variables, encompassing factors such as fast‐food consumption per week, hours of sleep per night, and physical activity per week. The exposures under scrutiny include energy drinks consumption (categorical), psychological indices (continuous), nutritional and lifestyle habits (both continuous and categorical), and gender (categorical). This comprehensive approach allows for a nuanced examination of the associations between these variables among Israeli‐Arab adolescents, offering valuable insights into the interplay of energy drinks consumption, anxiety and well‐being, nutritional, and lifestyle factors in this population.

*T*‐tests and ANOVA were used due to prior examinations showing that variables under investigation approximate normality. To test for possible differences in anxiety and well‐being reports between energy drinks consumers and nonconsumers, independent *t*‐tests were performed. ANOVAs were performed to examine differences in energy drinks consumption, psychological indices, and nutritional and lifestyle habits. Post hoc analyses were conducted using Students’ *t*‐tests. Chi‐square tests were conducted on nutritional and lifestyle habit indices (fast‐food consumption per week, hours of sleep per night, and engagement in physical activity per week) between genders. To test for possible correlations between anxiety and well‐being levels and hours of sleep per night, a Spearman coefficient analysis was performed among energy drinks consumers and nonconsumers. Other gender‐related associations were also examined.

All statistical analyses of the participants’ questionnaire answers were performed using SPSS 23.0. To test the degree to which the data support the associations, we used JASP 0.17.1. Graphs and visualization were conducted using GraphPad Prism version 8.0.0 for Windows, GraphPad Software, www.graphpad.com.

### Ethical considerations

The Helsinki Committee at the Saint Vincent de Paul Hospital approved and conducted this study. Before participation, participants approved their participation, and written informed consent was obtained from their parents in person. Participation was voluntary.

## RESULTS

One hundred and seventy subjects were recruited for the study, with the final participation of 114 volunteers. Of the initial 170 participants recruited between August 2019 and March 2020, 20 subjects were excluded due to an inability to track measures during the COVID‐19 quarantines; 36 others were excluded due to incomplete questionnaires. Thus, the final sample consisted of 114 participants (59 females, 99 Muslims) (see Table 1). Using a self‐report questionnaire regarding the nutritional status and ED energy drinks consumption, we obtained two groups of adolescents; one group reported consuming energy drinks at least once a week (*n* = 45); the other did not (*n* = 69).

**TABLE 1:** Characteristics of 114 participants from northern Israel between 2019 and 2020, including gender, religion, father and mother education level, age, weight classification, waist‐height ratio classification and frequency of energy drinks consumption per week (data are presented by number of subjects and percentages; mean values with standard deviations and ranges).

		Frequency *n* (%)
**Gender**		
	*Male*	55 (48)
	*Female*	59 (52)
**Religion**		
	*Muslim*	99 (86)
	*Christian*	15 (14)
**Father's education level**		
	*Incomplete primary and Junior High School*	35 (31)
	*Primary and junior high school*	38 (34)
	*Academic (B.A., M.A., Ph.D.)*	17 (15)
	*Nonacademic profession*	8 (7)
	*Other*	6 (5)
	*Not Known*	10 (8)
**Mother's education level**		
	*Incomplete primary and Junior High School*	21 (19)
	*Primary and junior high school*	46 (41)
	*Academic (B.A., M.A., Ph.D.)*	29 (25)
	*Nonacademic profession*	7 (6)
	*Other*	5 (4)
	*Not Known*	6 (5)

		MEAN	SD	Range
**Age (years)**				
	*Male*	14.18	1.74	12.00–17.11
	*Female*	15.02	1.59	12.01–17.11
		**Frequency *n* (%)**		
**Weight classification**				
	*Normal weight*	68 (60)		
	*Overweight*	26 (23)		
	*Obese*	20 (17)		
**Waist‐height ratio classification**				
	*Normal*	84 (74)		
	*High risk*	30 (26)		

	Once (1)	2–3	4–5	6 or more
**Consumption of energy drinks per week *(n =* *45), n (%)***	20 (44)	11 (24)	9 (20)	5 (12)

A preliminary analysis of participants’ demographics and anthropomorphic indices was performed, and the frequency of energy drinks consumption per week was reported (see Table 1). Gender proportion, waist‐to‐height ratio, and weight distribution between groups (energy drinks consumers and nonconsumers) did not differ. Therefore, neither waist‐to‐height ratio nor BMI was included in the statistical analyses as covariates (data not shown).

### Energy drinks consumption is associated with a higher anxiety score

**FIGURE 1:**
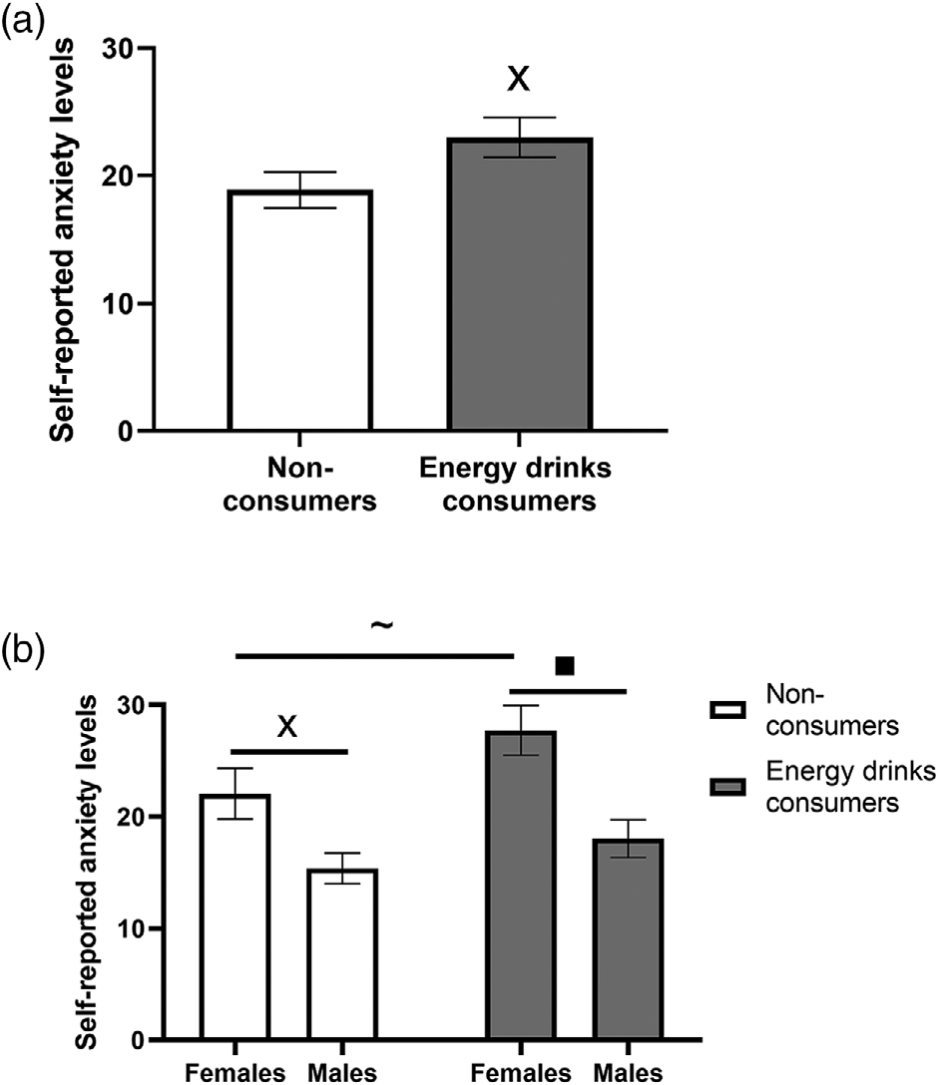
Energy drinks consumption is associated with a higher anxiety score: (a) Results are presented as mean ± SEM; energy drinks consumers (*n* = 45), nonconsumers (*n* = 69), ×*p* < 0.05; (b) results are presented as mean ± SEM, female energy drinks consumers (*n* = 23), male energy drinks consumers (*n* = 22), female nonconsumers (*n* = 36), male nonconsumers (*n* = 33), ■*p* < 0.01;×*p* < 0.05, ∼*p* = 0.07.

To test for possible differences in self‐reports of anxiety between energy drinks consumers and nonconsumers, an independent Student's *t*‐test was performed. Figure 1a depicts the significant difference found for the reported anxiety levels between the two groups (*p* = 0.05). Specifically, energy drinks consumers reported higher anxiety levels (mean = 23.00, SEM = 1.56) than nonconsumers (mean = 18.86, SEM = 1.41).

Univariate ANOVA was performed to test for possible differences in anxiety between the genders among energy drinks consumers and nonconsumers. A significant effect was found for gender (*p* < 0.01, partial *η* ^2^ = 0.13) and for energy drinks consumption (*p* = 0.04, partial *η* ^2^ = 0.04), but not for the gender × energy drinks consumption interaction]*p* = 0.46]. Specifically, females and energy drinks consumers reported higher levels of anxiety than males and nonconsumers (data not shown). Additional post hoc comparisons conducted by Student's *t*‐tests revealed that among energy drinks consumers, females reported significantly higher anxiety levels (mean = 27.74, SEM = 2.22) than males (mean = 18.04, SEM = 1.67)—(*p* < 0.01). A similar effect was also found for nonconsumers, with females reporting significantly higher anxiety levels (mean = 22.06, SEM = 2.27) compared to males (mean = 15.39, SEM = 1.37)—(*p* = 0.01). Among males, no significant difference was observed in the reported anxiety levels between energy drinks consumers and nonconsumers (*p = *0.23). In females, a marginal difference was found in the reported anxiety levels between energy drinks consumers and nonconsumers (*p = *0.07), with energy drinks consumers reporting slightly higher levels of anxiety (mean = 27.74, SEM = 2.22) compared to nonconsumers (mean = 22.06, SEM = 2.27) (Figure 1b).

### Energy drinks consumption is associated with a lower well‐being score

**FIGURE 2:**
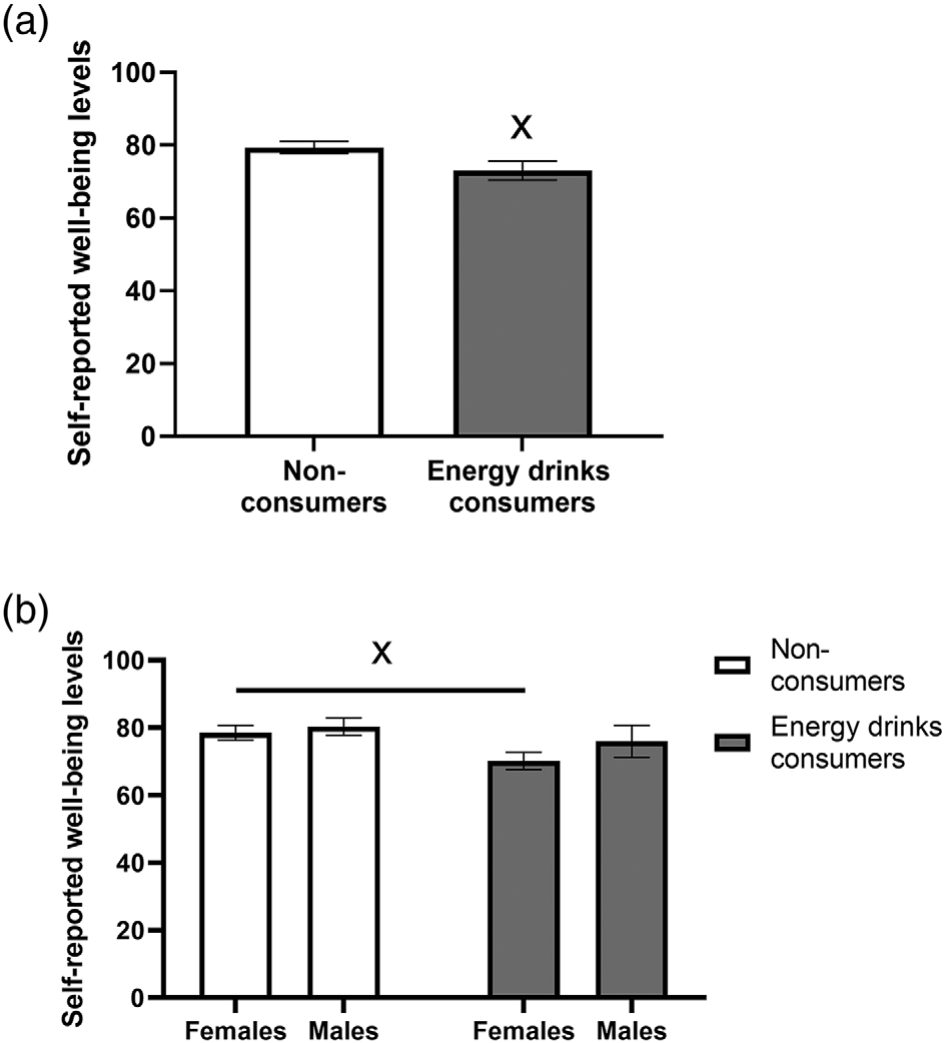
Energy drinks consumption is associated with a lower well‐being score: (a) Results are presented as mean ± SEM; energy drinks consumers (*n* = 45), nonconsumers (*n* = 69), ×*p* < 0.05; (b) results are presented as mean ± SEM, female energy drinks consumers (*n* = 23), male energy drinks consumers (*n* = 22), female nonconsumers (*n* = 36), male nonconsumers (*n* = 33), ×*p* < 0.05.

An independent Student's *t*‐test was performed to test for possible differences in self‐reported well‐being between energy drinks consumers and nonconsumers. Figure 2a depicts the significant difference found for well‐being reports between the two groups (*p* = 0.03). Specifically, energy drinks consumers reported lower levels of well‐being (mean = 73.02, SEM = 2.64) than nonconsumers (mean = 79.37, SEM = 1.67).

To further elucidate this effect, univariate ANOVA was performed to test for possible differences between the genders among energy drinks consumers and nonconsumers. A significant effect was found for energy drinks consumption (*p* = 0.04, partial *η* ^2^ = 0.04) but not for gender (*p = *0.20), or for the gender × energy drinks consumption interaction (*p* = 0.49) (data not shown). Additional post hoc comparisons conducted by using Student's *t*‐tests revealed that only female energy drinks consumers reported lower well‐being levels (mean = 70.17, SEM = 2.57) than female nonconsumers (mean = 78.51, SEM = 2.21)—(*p* = 0.01). No differences were observed among males between energy drinks consumers and nonconsumers in well‐being reports (Figure 2b).

### Energy drinks consumption effects on lifestyle and nutritional habits

We also tested for possible differences between the genders among energy drinks consumers and nonconsumers in their nutritional and lifestyle habits (fast‐food consumption per week, engagement in physical activity per week, and hours of sleep per night).

**FIGURE 3:**
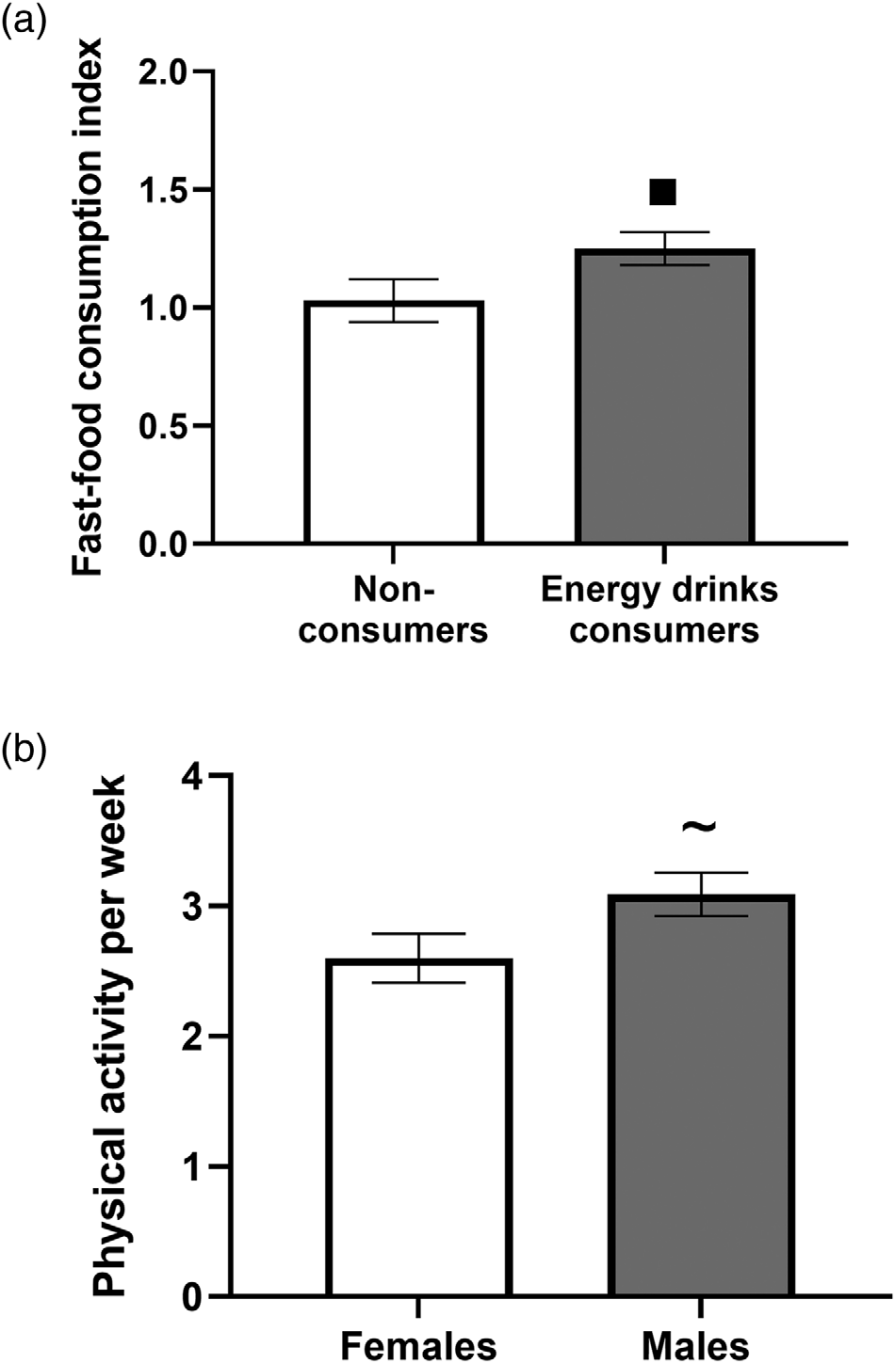
Energy drinks consumption affects lifestyle and nutritional habits. (a) Fast‐food consumption index by energy drinks consumption. Results are presented as mean ± SEM, energy drinks consumers (*n* = 45), nonconsumers (*n* = 69), ■*p* < 0.01. (b) Participation in physical activity per week by gender. Results are presented as mean ± SEM, males (*n* = 55), females (*n* = 59), ∼*p* = 0.07.

The proportion of fast‐food consumption per week (as indicated by the fast‐food consumption index) significantly differed between energy drinks consumers and nonconsumers [*χ* ^2^ (1) = 8.18, *p <* 0.01]. Energy drinks consumers ate significantly more fast food per week (mean = 1.25, SEM = 0.07) than nonconsumers (mean = 1.03, SEM = 0.09) (Figure 3a). Univariate ANOVA indicated a marginal gender effect on physical activity among energy drinks consumers and nonconsumers (*p* = 0.07, partial *η* ^2^ = 0.03). Post hoc tests revealed slightly higher physical activity in males compared to females (*p* = 0.078) (Figure 3b). A Spearman correlation analysis found a significant association between anxiety levels and hours of sleep per night only among energy drinks consumers (*r*_s_ = 0.352, *p* = 0.018), explicitly indicating that higher anxiety correlates with more sleep. Further examination by gender revealed a positive correlation for female energy drinks consumers (*r*_s_ = 0.574, *p* < 0.01), contrasting with nonconsumers and male groups (*p* > 0.05) (data not shown).

Quantifying the data's support for this association, we conducted nondirectional Bayesian Kendall correlations using JASP software (Version 0.17.1) due to the small sample size and the lack of a significant interaction between energy drinks consumption and gender in previous analyses. Strong evidence of a positive correlation between anxiety levels and hours of sleep per night among female energy drinks consumers emerged (Bayes factor of 19.54). The Kendall tau correlation coefficient was 0.461, with a 95% credible interval of (0.133, 0.667), indicating a moderate positive association. Conversely, weak evidence against the null hypothesis was found for female nonconsumers, male energy drinks consumers, and male nonconsumers (Bayes factors of 0.315, 0.343, and 0.395, respectively), with Kendall tau coefficients of 0.103, 0.106, and 0.132, respectively. The 95% credible intervals suggested a broad range of plausible values for the correlation coefficient (Figure S1A–D).

## DISCUSSION

Our results revealed that energy drinks consumption is associated with reports of elevated anxiety and lower well‐being at the cross‐sectional levels. This aligns with previous findings where energy drinks consumption was associated with increased anxiety in young adult males [32]. Thus, a study on high school students found that energy drinks consumption was significantly associated with lower subjective well‐being, worse symptoms of depression, and more emotional disturbances [21]. However, the gender effect in the reports found in our sample has not been reported previously. Our results revealed a gender difference in anxiety levels regardless of energy drinks consumption; female energy drinks consumers and nonconsumers reported significantly higher anxiety levels than their male counterparts. These results fit well with those reported in the scientific literature [33, 34]. Moreover, energy drinks consumption was associated with lower well‐being scores only in female consumers versus female nonconsumers. Importantly, no interaction was found between energy drinks consumption and gender for anxiety and well‐being levels.

Research on gender differences, energy drinks consumption, and mental health outcomes has yielded different results. Young adult males who transitioned from non‐energy drinks consumers to energy drinks consumers experienced an average increase in depression, anxiety, and stress, whereas this was not observed among females [35]. Furthermore, studies reported that boys tend to consume more energy drinks than girls and that this consumption was associated with risky behaviors, anxiety, depression, and poor academic performance [36, 37]. These findings suggest that although there may be direct gender differences in the mental health outcomes of energy drinks consumption, gender disparities in consumption patterns may also indirectly impact mental health.

Caffeine in energy drinks is associated with these affective psychological symptoms. Several caffeine‐withdrawal symptoms have been reported, including headache, fatigue, sleepiness, dysphoric mood (e.g., feeling miserable and decreased well‐being), decreased cognitive performance, depression, irritability, and nausea/vomiting [38]. Among adolescents and young adults, caffeine consumption is positively related to attention deficit hyperactivity disorder symptoms [39] and sleep disturbances [40]. To further elucidate the association between energy drinks consumption and the reported anxiety and well‐being levels, we examined nutritional and lifestyle habits (fast‐food consumption per week, hours of sleep per night, and physical activity per week). A significant association was found between energy drinks consumption and the frequency of fast‐food consumption per week. Such an association was also reported in a cross‐sectional study on 8–10‐year‐olds, showing that energy drinks consumers were likelier to consume high energy‐dense fast foods versus nonconsumers [41]. Similarly, a recent study reported a significant association between frequent fast foods, energy drinks, and convenience food consumption and newly diagnosed atopic dermatitis in adolescents [42]. These findings further strengthen the current results.

A gender difference was also found in the frequency of weekly physical activity, with males engaging in more physical activity than females. However, this finding was not related to energy drinks consumption. In line with our results, several other studies found no association between energy drinks consumption and the level of physical activity [43, 44]. However, one study reported an inverse association between energy drinks consumption and the frequency of physical activity [45]. Such contradictory results may reflect cultural differences, methodological differences such as the number of participants, and differences in assessing physical activity and energy drinks consumption frequency.

Last, we also observed a difference in the association between anxiety levels and hours of sleep per night among energy drinks consumers. The link between sleep and energy drinks is consistent and has been represented in previous studies [46, 47]. Some have shown that energy drinks consumption is negatively associated with sleep duration in both genders [48, 49]. Others found that energy drinks consumption was associated with reduced sleep duration, especially among boys [41]. However, none of these papers examined the consumption of energy drinks concerning psychological indices, particularly anxiety. Our results align with previous reports showing that sleep quantity is associated with energy drinks consumption [41, 48, 49]. Yet, in contrast to previous findings, ours are in the opposite direction. We found that energy drinks consumers reported more hours of sleep per night, and this was evident only among female responders, who also reported higher anxiety levels. Future studies should further examine this finding and, in addition, examine not only sleep quantity but also its quality.

Although our findings align with existing literature, the gender difference in anxiety levels among both energy drinks consumers and nonconsumers in our sample represents a novel observation. The association between caffeine in energy drinks and affective psychological symptoms, as well as the correlation between energy drinks consumption, fast‐food intake, and well‐being, is consistent with prior research. Nevertheless, the gender difference in physical activity, unrelated to energy drinks consumption, diverges from some studies, emphasizing the need for cautious interpretation. Furthermore, our findings on the association between anxiety levels and sleep duration among energy drinks consumers, particularly females, present a unique perspective compared to the current literature, highlighting the importance of further exploration and consideration of sleep quality in future studies.

### Strengths and limitations

Self‐reported questionnaires could introduce a potential bias. To mitigate their impact, the questionnaires used to assess mental health outcomes were for school‐aged children and adolescents, making the results more valid and representative of this age group. Completing the questionnaires independently, without the presence of the parents, aimed to ensure that the adolescents felt more comfortable providing honest and accurate answers without any influence. Furthermore, as adolescence is challenging for implementing nutritional and lifestyle habit changes, we chose to focus our research on this age group. The obtained data will broaden the scientific knowledge base on developing populations and lead to a possible change in the associations between nutritional and lifestyle habits and psychological symptoms by applying a pre‐ to post‐nutritional intervention. Moreover, our study is the first to combine the psychological, nutritional, and lifestyle facets of these associations between male and female adolescents together.

The current study has some limitations. First, due to the COVID‐19 pandemic, we had to stop recruiting, and thus, our analysis only covered results from 114 adolescents. Further, recall and reporting bias with self‐reported data are risks. Most of the participants in this study were Muslim adolescents from Arab society in Israel. As energy drinks are considered to be drinks that can be mixed with alcohol, we assume that some of the participants felt uncomfortable reporting the absolute frequency of consuming these products. This could lead to an underestimation of the number of adolescents consuming energy drinks and the specific frequency of energy drinks consumption. Such underestimation could impact the gender‐related results in our study. Moreover, we collected data from a small convenience sample and conducted simple and repeated bivariate analyses; this might limit the practical implications drawn from the study. The study's cross‐sectional nature further restricts our ability to infer causal relationships between variables. Additionally, the study does not explicitly adjust for confounders, which might lead to less accurate conclusions about the true impact of energy drinks consumption on mental health. It is challenging to attribute observed differences solely to energy drinks without considering confounders, emphasizing the need for cautious interpretation.

### Future directions

Future research could explore the nuanced gender differences in anxiety levels among energy drinks consumers, shedding light on potential underlying factors. Investigating the impact of energy drinks consumption on sleep quality beyond quantity could provide a more comprehensive understanding of its association with mental health. Additionally, a longitudinal design would facilitate the exploration of causal relationships between energy drinks consumption and psychological outcomes, addressing limitations associated with the cross‐sectional nature of the current study. Considering diverse populations and potential cultural influences on reporting habits would further enrich the understanding of the psychological implications of energy drinks consumption.

## CONCLUSION

We found that gender and energy drinks consumption mediate psychological status. Energy drinks consumers reported a lower well‐being index and a higher anxiety index than nonconsumers. Energy drinks consumers ate significantly more fast food per week than nonconsumers. Energy drinks consumption was also linked to sleep. National public health authorities must consider the association between energy drinks consumption among adolescents and psychological aspects from the perspective of a possible.

## AUTHOR CONTRIBUTIONS

*Formulated the research questions; designed the study and carried it out; was responsible for the adolescent's recruitment; questionnaires data collection; assessing anthropometric measurements; analyzing and interpreting the data; writing and drafting the article*: Lili Nimri. *Participated in designing the study; was responsible for staff organization; supervised the study process; participated in data collection; adolescents’ recruitment; interpreting the data and drafting the article*: Bshara Mansour. *Participated in analyzing and interpreting the data*: Amir Benhos. *Participated in adolescents’ recruitment; data collection and assessing anthropometric measurements*: Abdallah Banna. *Participated in adolescents’ recruitment and data collection*: Elias Nasrallah, Marwan Sackran, and Ahlam Abu Ahmad. *Participated in drafting the article*: Ziv Ardi. *Formulated the research questions; designed the study and carried it out; was responsible for analyzing and interpreting the data; writing and drafting the article*: Omer Horovitz. All authors read and approved the submitted version of the manuscript.

## CONFLICT OF INTEREST STATEMENT

The authors declare no conflicts of interest.

## ETHICS STATEMENT

This study was conducted according to the guidelines laid down in the Declaration of Helsinki, and all procedures involving research study participants were approved by the Helsinki Committee of Saint Vincent de Paul Hospital. Participants gave their consent and written informed consent was obtained from their parents.

## Supporting information

Supporting Information

Supporting Information

## Data Availability

The datasets generated and/or analyzed during the current study are not publicly available due their containing information that could compromise the privacy of research participants but are available from the corresponding author on reasonable request.

## References

[1] Kaplan BJ , Rucklidge JJ , Romijn A , et al. The emerging field of nutritional mental health: inflammation, the microbiome, oxidative stress, and mitochondrial function. Clin Psychol Sci. 2015;3:964‐980.

[2] Firth J , Torous J , Nicholas J , et al. The efficacy of smartphone‐based mental health interventions for depressive symptoms: a meta‐analysis of randomized controlled trials. World Psychiatry. 2017;16:287‐298. 28941113 10.1002/wps.20472 PMC5608852

[3] Firth J , Torous J , Nicholas J , et al. Can smartphone mental health interventions reduce symptoms of anxiety? A meta‐analysis of randomized controlled trials. J Affect Disord. 2017;218:15‐22. 28456072 10.1016/j.jad.2017.04.046

[4] Jacka FN , Cherbuin N , Anstey KJ , et al. Dietary patterns and depressive symptoms over time: examining the relationships with socioeconomic position, health behaviours and cardiovascular risk. PLoS ONE. 2014;9:e87657. 24489946 10.1371/journal.pone.0087657 PMC3906192

[5] Quirk SE , Williams LJ , O'Neil A , et al. The association between diet quality, dietary patterns and depression in adults: a systematic review. BMC Psychiatry. 2013;13:175. 23802679 10.1186/1471-244X-13-175 PMC3706241

[6] Jacka FN , Pasco JA , Mykletun A , et al. Association of western and traditional diets with depression and anxiety in women. Am J Psychiatry. 2010;167:305‐311. 20048020 10.1176/appi.ajp.2009.09060881

[7] Owen L , Corfe B . The role of diet and nutrition on mental health and wellbeing. Proc Nutr Soc. 2017;76:425‐426. 28707609 10.1017/S0029665117001057

[8] Muscaritoli M . The impact of nutrients on mental health and well‐being: insights from the literature. Front Nutr. 2021;8:656290. 33763446 10.3389/fnut.2021.656290 PMC7982519

[9] Adan RAH , Van Der Beek EM , Buitelaar JK , et al. Nutritional psychiatry: towards improving mental health by what you eat. Eur Neuropsychopharmacol. 2019;29:1321‐1332. 31735529 10.1016/j.euroneuro.2019.10.011

[10] Potischman N , Weed DL . Causal criteria in nutritional epidemiology. Am J Clin Nutr. 1999;69:1309S‐1314S. 10359231 10.1093/ajcn/69.6.1309S

[11] Baron‐Epel O , Kaplan G . Can subjective and objective socioeconomic status explain minority health disparities in Israel? Soc Sci Med. 2009;69:1460‐1467. 19765878 10.1016/j.socscimed.2009.08.028

[12] Kabha A , Enav T , Manor N , et al. Energy drinks Arab children 2011 survey. In: Health Surveys. The Israeli Ministry of Health; 2011. Accessed 3 September 2021. https://www.health.gov.il/UnitsOffice/ICDC/Health_Surveys/Pages/Energy_drinks_among_arb.aspx

[13] Magnezi R , Bergman LC , Grinvald‐Fogel H , et al. A survey of energy drink and alcohol mixed with energy drink consumption. Isr J Health Policy Res. 2015;4:55. 26629327 10.1186/s13584-015-0052-5 PMC4665319

[14] The Israeli Ministry of Health . Mabat_youth_2015‐2016—the Israeli Ministry of Health. The Israeli Ministry of Health. 2017.(Accessed 4 September 2021) https://www.health.gov.il/publicationsfiles/mabat_kids2_11_2015‐2016‐eng.pdf</bib>

[15] Peng W , Goldsmith R , Berry EM . Demographic and lifestyle factors associated with adherence to the Mediterranean diet in relation to overweight/obesity among Israeli adolescents: findings from the Mabat Israeli national youth health and nutrition survey. Public Health Nutr. 2017;20:883‐892. 27829478 10.1017/S1368980016002779 PMC10261575

[16] Chernichovsky D , Bsharat B , Brial A , et al. TAUB Center Report‐ Health of the Arab Israeli Population. TAUB Center for Social Policy Studies in Israel. 2017. Accessed 3 September 2021. https://www.taubcenter.org.il/wp‐content/uploads/2020/12/healthofthearabisraelipopulation.pdf

[17] Li Y , Lv M‐R , Wei Y‐J , et al. Dietary patterns and depression risk: a meta‐analysis. Psychiatry Res. 2017;253:373‐382. 28431261 10.1016/j.psychres.2017.04.020

[18] Gunja N , Brown JA . Energy drinks: health risks and toxicity. Med J Aust. 2012;196:46‐49. 22256934 10.5694/mja11.10838

[19] Mansour B , Amarah W , Nasralla E , et al. Energy drinks in children and adolescents: demographic data and immediate effects. Eur J Pediatr. 2019;178:649‐656. 30770983 10.1007/s00431-019-03342-7

[20] Koivusilta L , Kuoppamäki H , Rimpelä A . Energy drink consumption, health complaints and late bedtime among young adolescents. Int J Public Health. 2016;61:299‐306. 26888471 10.1007/s00038-016-0797-9

[21] Utter J , Denny S , Teevale T , et al. Energy drink consumption among New Zealand adolescents: associations with mental health, health risk behaviours and body size. J Paediatr Child Health. 2018;54:279‐283. 28905482 10.1111/jpc.13708

[22] Dawodu A , Cleaver K . Behavioural correlates of energy drink consumption among adolescents: a review of the literature. J Child Health Care. 2017;21:446‐462. 29110525 10.1177/1367493517731948

[23] Ali F , Rehman H , Babayan Z , et al. Energy drinks and their adverse health effects: a systematic review of the current evidence. Postgrad Med. 2015;127:308‐322. 25560302 10.1080/00325481.2015.1001712

[24] de Onis M , Onyango AW , Borghi E , et al. Development of a WHO growth reference for school‐aged children and adolescents. Bull World Health Organ. 2007;85:660‐667. 18026621 10.2471/BLT.07.043497 PMC2636412

[25] Sharma AK , Metzger DL , Daymont C , et al. LMS tables for waist‐circumference and waist‐height ratio z‐scores in children aged 5–19 y in NHANES III: association with cardio‐metabolic risks. Pediatr Res. 2015;78:723‐729. 26331767 10.1038/pr.2015.160

[26] Birmaher B , Brent DA , Chiappetta L , et al. Psychometric properties of the screen for child anxiety related emotional disorders (SCARED): a Replication Study. J Am Acad Child Adolesc Psychiatry. 1999;38:1230‐1236. 10517055 10.1097/00004583-199910000-00011

[27] Muris P , Dreessen L , Bögels S , et al. A questionnaire for screening a broad range of DSM‐defined anxiety disorder symptoms in clinically referred children and adolescents. J Child Psychol Psychiatry. 2004;45:813‐820. 15056312 10.1111/j.1469-7610.2004.00274.x

[28] Tomyn AJ , Norrish JM , Cummins RA . The subjective wellbeing of indigenous Australian adolescents: validating the personal wellbeing index‐school children. Soc Indic Res. 2013;110:1013‐1031.

[29] Attila S , Çakir B . Energy‐drink consumption in college students and associated factors. Nutrition. 2011;27:316‐322. 20579846 10.1016/j.nut.2010.02.008

[30] Man CS , Hock LK , Ying CY , et al. Is fast‐food consumption a problem among adolescents in Malaysia? An analysis of the national school‐based nutrition survey, 2012. J Health Popul Nutr. 2021;40:31. 34271986 10.1186/s41043-021-00254-x PMC8285850

[31] Kumar B , Robinson R , Till S . Physical activity and health in adolescence. Clin Med. 2015;15:267‐272. 10.7861/clinmedicine.15-3-267 PMC4953112 26031978

[32] Trapp GSA , Allen K , O'Sullivan TA , et al. Energy drink consumption is associated with anxiety in Australian young adult males. Depress Anxiety. 2014;31:420‐428. 24019267 10.1002/da.22175

[33] Ohannessian CM , Milan S , Vannucci A . Gender differences in anxiety trajectories from middle to late adolescence. J Youth Adolesc. 2017;46:826‐839. 27889856 10.1007/s10964-016-0619-7 PMC5815170

[34] Lewinsohn PM , Gotlib IH , Lewinsohn M , et al. Gender differences in anxiety disorders and anxiety symptoms in adolescents. J Abnorm Psychol. 1998;107:109‐117. 9505043 10.1037//0021-843x.107.1.109

[35] Kaur S , Christian H , Cooper MN , et al. Consumption of energy drinks is associated with depression, anxiety, and stress in young adult males: evidence from a longitudinal cohort study. Depress Anxiety. 2020;37:1089‐1098. 32845046 10.1002/da.23090

[36] Silva‐Maldonado P , Arias‐Rico J , Romero‐Palencia A , et al. Consumption patterns of energy drinks in adolescents and their effects on behavior and mental health: a systematic review. J Psychosoc Nurs Ment Health Serv. 2022;60:41‐47. 10.3928/02793695-20210818-04 34432594

[37] Visram S , Cheetham M , Riby DM , et al. Consumption of energy drinks by children and young people: a rapid review examining evidence of physical effects and consumer attitudes. BMJ Open. 2016;6:e010380. 10.1136/bmjopen-2015-010380 PMC5073652 27855083

[38] Juliano LM , Griffiths RR . A critical review of caffeine withdrawal: empirical validation of symptoms and signs, incidence, severity, and associated features. Psychopharmacology (Berl). 2004;176:1‐29. 15448977 10.1007/s00213-004-2000-x

[39] Martin CA , Cook C , Woodring JH , et al. Caffeine use: association with nicotine use, aggression, and other psychopathology in psychiatric and pediatric outpatient adolescents. Sci World J. 2008;8:512‐516. 10.1100/tsw.2008.82 PMC3176831 18516472

[40] Bryant Ludden A , Wolfson AR . Understanding adolescent caffeine use: connecting use patterns with expectancies, reasons, and sleep. Health Educ Behav Off Publ Soc Public Health Educ. 2010;37:330‐342. 10.1177/1090198109341783 19858312

[41] Almulla AA , Faris MA‐IE . Energy drinks consumption is associated with reduced sleep duration and increased energy‐dense fast foods consumption among school students: a cross‐sectional study. Asia Pac J Public Health. 2020;32:266‐273. 32508133 10.1177/1010539520931351

[42] Cho SI , Lee H , Lee DH , et al. Association of frequent intake of fast foods, energy drinks, or convenience food with atopic dermatitis in adolescents. Eur J Nutr. 2020;59:3171‐3182. 31822988 10.1007/s00394-019-02157-4

[43] Faris AE , Epuru S , Al‐Shimmari S , Al‐Shimmar E. Alarming high levels of energy drinks consumption among school children in hail, Northern of Saudi Arabia. Int J Child Health Nutr. 2015;4:1‐13.

[44] Larson N , DeWolfe J , Story M , et al. Adolescent consumption of sports and energy drinks: linkages to higher physical activity, unhealthy beverage patterns, cigarette smoking, and screen media use. J Nutr Educ Behav. 2014;46:181‐187. 24809865 10.1016/j.jneb.2014.02.008 PMC4023868

[45] Degirmenci N , Fossum IN , Strand TA , et al. Consumption of energy drinks among adolescents in Norway: a cross‐sectional study. BMC Public Health. 2018;18:1391. 30567510 10.1186/s12889-018-6236-5 PMC6299924

[46] Park S , Lee Y , Lee JH . Association between energy drink intake, sleep, stress, and suicidality in Korean adolescents: energy drink use in isolation or in combination with junk food consumption. Nutr J. 2016;15:87. 27737671 10.1186/s12937-016-0204-7 PMC5064784

[47] Sampasa‐Kanyinga H , Hamilton HA , Chaput J‐P . Sleep duration and consumption of sugar‐sweetened beverages and energy drinks among adolescents. Nutr Burbank Los Angel Cty Calif. 2018;48:77‐81. 10.1016/j.nut.2017.11.013 29469025

[48] Kaldenbach S , Leonhardt M , Lien L , et al. Sleep and energy drink consumption among Norwegian adolescents—a cross‐sectional study. BMC Public Health. 2022;22:534. 35303832 10.1186/s12889-022-12972-w PMC8932303

[49] Tomanic M , Paunovic K , Lackovic M , et al. Energy drinks and sleep among adolescents. Nutrients. 2022;14:3813. 36145187 10.3390/nu14183813 PMC9502542

