# Supplementary material for: Energy drink consumption among Israeli‐Arab adolescents: Gender differences in anxiety and well‐being
Source: Public Health Chall. 2024 Jul 17;3(3):e187. doi: 10.1002/puh2.187 (PMC12039572; doi:10.1002/puh2.187)
Supplement: Supplementary file 1 — Supporting Information [file PUH2-3-e187-s002.docx]

| **Supplementary** **Figur S1A**  **Association between Anxiety levels and Sleep hours per night among female ED consumers** | |
| --- | --- |
| \| **Bayesian Kendall's Tau Correlations** \| \| \| \| \| \| \| \| \| --- \| --- \| --- \| --- \| --- \| --- \| --- \| --- \| \| **Variable** \| \|  \| \| **Anxiety** \| \| **Sleep** \| \| \| 1. Anxiety \|  \| Kendall's tau \|  \| — \|  \|  \|  \| \|  \|  \| BF₁₀ \|  \| — \|  \|  \|  \| \| 2. Sleep \|  \| Kendall's tau \|  \| 0.461 \|  \| — \|  \| \|  \|  \| BF₁₀ \|  \| 19.545 \|  \| — \|  \| \|  \| \| \| \| \| \| \| \| | Prior and Posterior 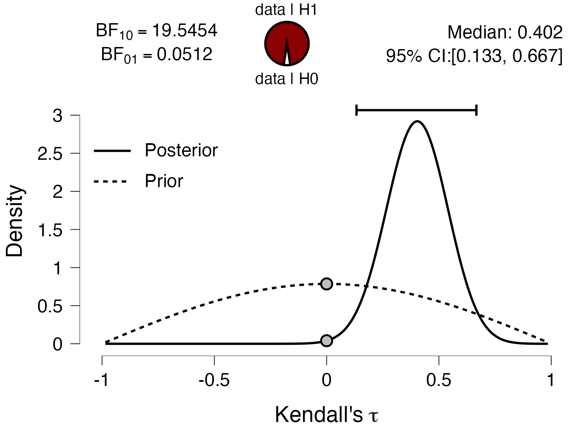 |
| **Supplementary** **Figure S1B**  **Association between Anxiety levels and Sleep hours per night among female non-consumers** | |
| \| **Bayesian Kendall's Tau Correlations** \| \| \| \| \| \| \| \| \| --- \| --- \| --- \| --- \| --- \| --- \| --- \| --- \| \| **Variable** \| \|  \| \| **Anxiety** \| \| **Sleep** \| \| \| 1. Anxiety \|  \| Kendall's tau \|  \| — \|  \|  \|  \| \|  \|  \| BF₁₀ \|  \| — \|  \|  \|  \| \| 2. Sleep \|  \| Kendall's tau \|  \| 0.103 \|  \| — \|  \| \|  \|  \| BF₁₀ \|  \| 0.315 \|  \| — \|  \| \|  \| \| \| \| \| \| \| \| | Prior and Posterior 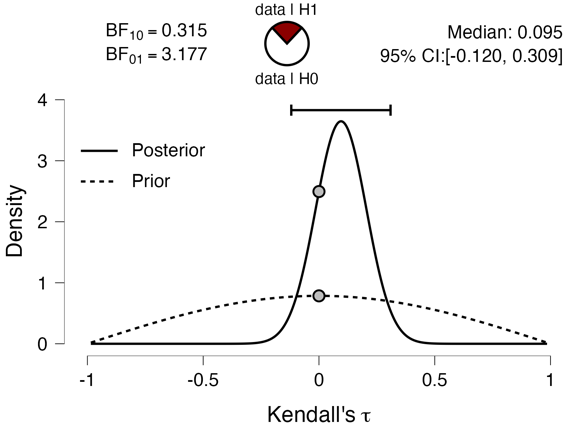 |

| **Supplementary** **Figure S1C**  **Association between Anxiety levels and Sleep hours per night among male ED consumers** | |
| --- | --- |
| \| **Bayesian Kendall's Tau Correlations** \| \| \| \| \| \| \| \| \| --- \| --- \| --- \| --- \| --- \| --- \| --- \| --- \| \| **Variable** \| \|  \| \| **Anxiety** \| \| **Sleep** \| \| \| 1. Anxiety \|  \| Kendall's tau \|  \| — \|  \|  \|  \| \|  \|  \| BF₁₀ \|  \| — \|  \|  \|  \| \| 2. Sleep \|  \| Kendall's tau \|  \| 0.106 \|  \| — \|  \| \|  \|  \| BF₁₀ \|  \| 0.343 \|  \| — \|  \| \|  \| \| \| \| \| \| \| \| | Prior and Posterior 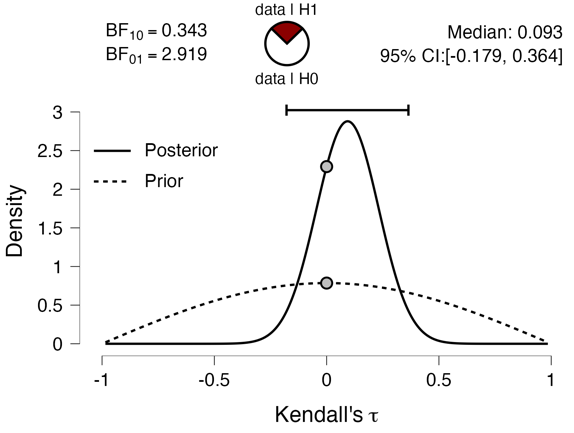 |
| **Supplementary** **Figure 1D**  **Association between Anxiety levels and Sleep hours per night among male non-consumers** | |
| \| **Bayesian Kendall's Tau Correlations** \| \| \| \| \| \| \| \| \| --- \| --- \| --- \| --- \| --- \| --- \| --- \| --- \| \| **Variable** \| \|  \| \| **Anxiety** \| \| **Sleep** \| \| \| 1. Anxiety \|  \| Kendall's tau \|  \| — \|  \|  \|  \| \|  \|  \| BF₁₀ \|  \| — \|  \|  \|  \| \| 2. Sleep \|  \| Kendall's tau \|  \| 0.132 \|  \| — \|  \| \|  \|  \| BF₁₀ \|  \| 0.395 \|  \| — \|  \| \|  \| \| \| \| \| \| \| \| | Prior and Posterior 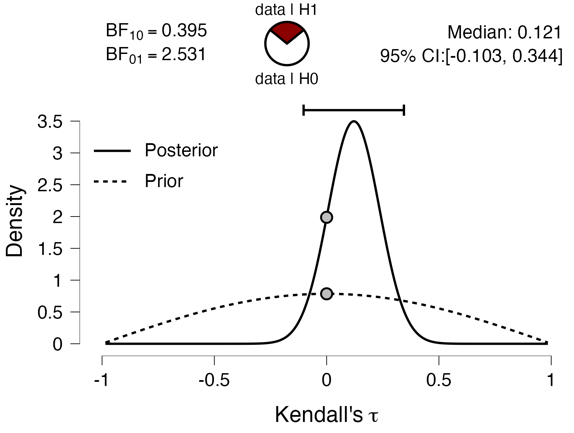 |

**S1 -** To quantify the degree to which the data supports this association, we further conducted nondirectional Bayesian Kendall correlations using JASP software (Version 0.17.1). This was done facing the small sample in each group, and the lack of significant interaction between ED consumption and gender observed in the previous analyses. **(A),** Our analysis found strong evidence of a positive correlation between anxiety levels and sleep hours per night among female ED consumers, with a Bayes factor of 19.54 in favor of the alternative hypothesis over the null hypothesis. Additionally, the Kendall tau correlation coefficient was 0.461, indicating a moderate positive association between anxiety levels and sleep hours per night. The 95% credible interval for the correlation coefficient was (0.133, 0.667), which suggests a high probability that the actual correlation coefficient falls within this range. Based on these findings, we can conclude that there is strong evidence of a moderate positive correlation between anxiety levels and sleep hours per night among these participants.

**(B-D),** In contrast, our analysis found weak evidence against the null hypothesis of no correlation between anxiety levels and sleep hours per night among female non-consumers **(B),** male ED consumers **(C),** and male non-consumers **(D),** with a Bayes factor of 0.315, 0.343, 0.395 (respectively) favoring the null hypothesis over the alternative hypothesis. The Kendall tau correlation coefficient was also 0.103, 0.106, and 0.132 (respectively). The 95% credible interval for the correlation coefficient was (-0.120, 0.309; -0.179, 0.364; -0.103, 0.344 - respectively), suggesting a wide range of plausible values for the correlation coefficient.
