# Supplementary material for: Energy drink consumption among Israeli‐Arab adolescents: Gender differences in anxiety and well‐being
Source: Public Health Chall. 2024 Jul 17;3(3):e187. doi: 10.1002/puh2.187 (PMC12039572; doi:10.1002/puh2.187)
Supplement: Supplementary file 2 — Supporting Information [file PUH2-3-e187-s001.docx]

| **Association between Anxiety levels and Sleep hours per night among female ED consumers** | |
| --- | --- |
| \| **Bayesian Kendall's Tau Correlations** \| \| \| \| \| \| \| \| \| --- \| --- \| --- \| --- \| --- \| --- \| --- \| --- \| \| **Variable** \| \|  \| \| **Anxiety** \| \| **Sleep** \| \| \| 1. Anxiety \|  \| Kendall's tau \|  \| — \|  \|  \|  \| \|  \|  \| BF₁₀ \|  \| — \|  \|  \|  \| \| 2. Sleep \|  \| Kendall's tau \|  \| 0.461 \|  \| — \|  \| \|  \|  \| BF₁₀ \|  \| 19.545 \|  \| — \|  \| \|  \| \| \| \| \| \| \| \| | Prior and Posterior 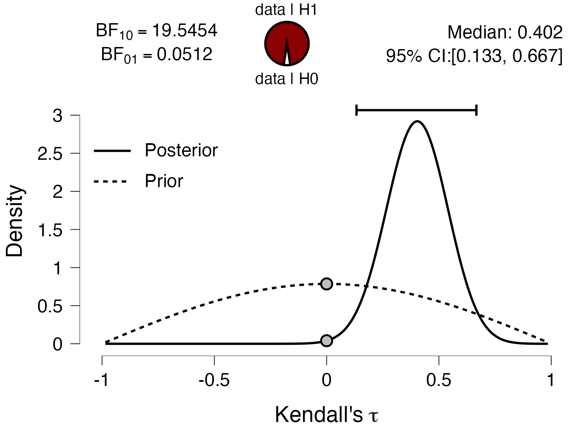 |
| **Association between Anxiety levels and Sleep hours per night among female non-consumers** | |
| \| **Bayesian Kendall's Tau Correlations** \| \| \| \| \| \| \| \| \| --- \| --- \| --- \| --- \| --- \| --- \| --- \| --- \| \| **Variable** \| \|  \| \| **Anxiety** \| \| **Sleep** \| \| \| 1. Anxiety \|  \| Kendall's tau \|  \| — \|  \|  \|  \| \|  \|  \| BF₁₀ \|  \| — \|  \|  \|  \| \| 2. Sleep \|  \| Kendall's tau \|  \| 0.103 \|  \| — \|  \| \|  \|  \| BF₁₀ \|  \| 0.315 \|  \| — \|  \| \|  \| \| \| \| \| \| \| \| | Prior and Posterior 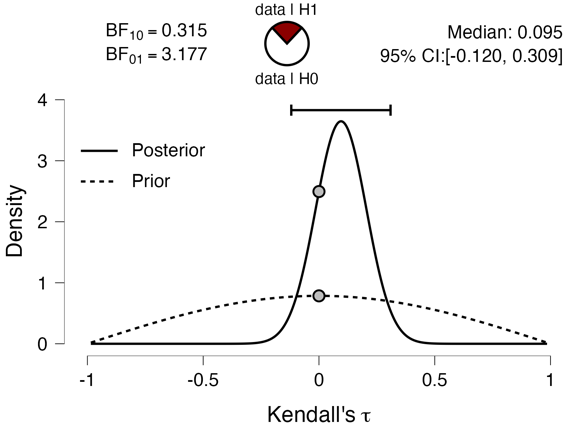 |

| **Association between Anxiety levels and Sleep hours per night among male ED consumers** | |
| --- | --- |
| \| **Bayesian Kendall's Tau Correlations** \| \| \| \| \| \| \| \| \| --- \| --- \| --- \| --- \| --- \| --- \| --- \| --- \| \| **Variable** \| \|  \| \| **Anxiety** \| \| **Sleep** \| \| \| 1. Anxiety \|  \| Kendall's tau \|  \| — \|  \|  \|  \| \|  \|  \| BF₁₀ \|  \| — \|  \|  \|  \| \| 2. Sleep \|  \| Kendall's tau \|  \| 0.106 \|  \| — \|  \| \|  \|  \| BF₁₀ \|  \| 0.343 \|  \| — \|  \| \|  \| \| \| \| \| \| \| \| | Prior and Posterior 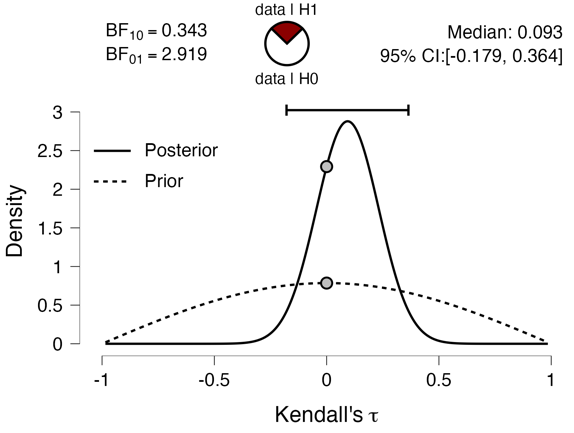 |
| **Association between Anxiety levels and Sleep hours per night among male non-consumers** | |
| \| **Bayesian Kendall's Tau Correlations** \| \| \| \| \| \| \| \| \| --- \| --- \| --- \| --- \| --- \| --- \| --- \| --- \| \| **Variable** \| \|  \| \| **Anxiety** \| \| **Sleep** \| \| \| 1. Anxiety \|  \| Kendall's tau \|  \| — \|  \|  \|  \| \|  \|  \| BF₁₀ \|  \| — \|  \|  \|  \| \| 2. Sleep \|  \| Kendall's tau \|  \| 0.132 \|  \| — \|  \| \|  \|  \| BF₁₀ \|  \| 0.395 \|  \| — \|  \| \|  \| \| \| \| \| \| \| \| | Prior and Posterior 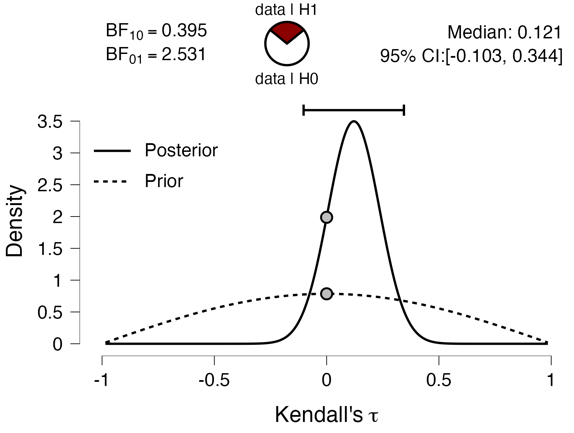 |
